# Determination of trace elements in ibuprofen drug products using microwave-assisted acid digestion and inductively coupled plasma-mass spectrometry

**DOI:** 10.1016/j.heliyon.2023.e23566

**Published:** 2023-12-10

**Authors:** Else Holmfred, Abdulla Alrijjal, C. Page Chamberlain, Katharine Maher, Stefan Stürup

**Affiliations:** a Department of Earth and Planetary Sciences, Stanford University, Stanford, CA, USA; b Department of Earth System Sciences, Stanford University, Stanford, CA, USA; c Department of Pharmacy, University of Copenhagen, Copenhagen, Denmark

**Keywords:** Ibuprofen, Pharmaceuticals, Trace elemental impurities, Acid digestion, ICP-MS, Analytical chemistry

## Abstract

Trace elements are found in most drugs as a result of the drug formulation and drug production methods. An inductively coupled plasma-mass spectrometry method for the determination of 24 trace elements (Mg, Ti, V, Cr, Mn, Cu, Fe, Co, Ni, Zn, As, Se, Mo, Ru, Rh, Pd, Ag, Cd, Sb, Ba, Ir, Pt, Au, and Pb) in solid ibuprofen tablets was established in relation to the ICH Q3D(R1) guideline, to evaluate the possibility of linking trace elemental profiles to drug formulation strategies, and to differentiate between drug products based on the trace elemental profiles. Ten European ibuprofen drug products were evaluated (n=3). The sample preparation was performed by microwave-assisted acid digestion using only 10 mg of homogenized sample and 900 μL of a mix of 65% HNO_3_, 37% HCl, and 30% H_2_O_2_. Solid residuals primarily composed of insoluble SiO_2_ excipients were removed by centrifugation. Only concentrations of Mg, Fe, Ti, Mn, Cr, and Ni were detected above the limits of detection and did not exceed the ICH Q3D(R1) guideline permitted daily exposure limits. The trace elemental profiles were evaluated through principal component analysis. Three principal components describing 96% of the variance were useful in grouping the ibuprofen drug products, and the detected trace elemental remnants could be related to drug formulation and drug production strategies. An in-house quality control material was used in lack of certified reference materials and was in combination with spike recoveries used for method validation. Good spike recoveries (94–119%) were obtained for all measured trace elements except Mg. Mg showed acceptable spike recoveries (75–155%) for mid and high-spike concentrations, but poor recoveries (30–223%) were detected with low spike concentrations in spike matrices containing high amounts of Mg. Overall, the method is suggested applicable for solid drugs containing insoluble SiO_2_ excipients and drugs comparable to ibuprofen.

## Highlights

• ICP-MS analysis of 24 trace elements in ten ibuprofen drug products.
• Microwave-assisted acid digestion used for extraction of trace elements.
• Principal component analysis used to distinguish between ibuprofen drug products.
• Lacking certified reference material, an in-house quality control material was used for validation.

## Graphical abstract

**Figure:**
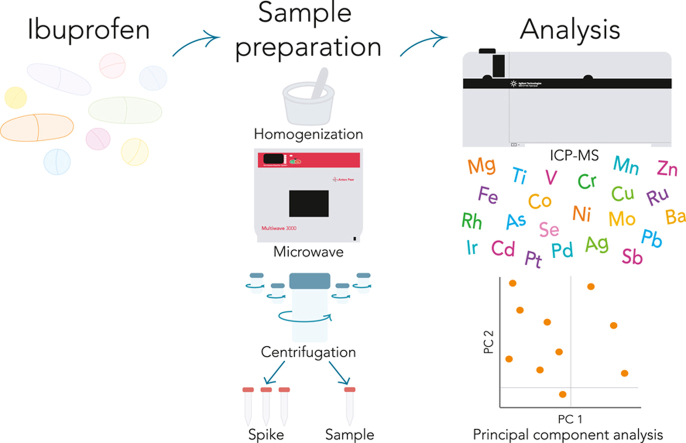

## 1 Introduction

Trace elements appear in most drugs as catalytic residues, impurity constituents of the active pharmaceutical ingredient (API) or excipients, and/or due to contamination from manufacturing equipment, production processes, or packaging [[1], [2], [3], [4], [5]]. Elemental impurities have no intended therapeutic effect and can be potentially toxic to humans. The presence of elemental impurities may even catalyze the decomposition of the API [3,6,7]. Consequently, the level of trace elements should be limited in the final drug product. In 2022, The International Council for Harmonization (ICH) of Technical Requirements for Pharmaceuticals for Human Use updated the Q3D(R2) guideline. The guideline identified 24 trace elements of potential toxicological concern and established permitted daily exposure limits accounting for the oral, parenteral, and inhalation route of administration (Li, V, Cr, Co, Ni, Cu, As, Se, Mo, Ru, Rh, Pd, Ag, Cd, Sn, Sb, Ba, Os, Ir, Pt, Au, Hg, Tl, and Pb) [5]. In addition, the ICH guideline highlights 10 trace elements (B, Na, Mg, Al, K, Ca, Mn, Fe, Zn, and W) that should be evaluated if present in the drug, but no permitted daily exposure limits have been established by ICH due to low inherent toxicity and/or variations in regional regulations [5]. The concern of toxicological effects related to excessive exposure to elemental impurities is also considered in various pharmacopeias [8,9].

Afterwards, the United States Pharmacopoeia (USP) updated the *<232> Elemental Impurities - Limits* monographs and adapted the ICH Q3D(R2) guideline for oral, parenteral, and inhalation permitted daily exposure limits for all 24 trace elements of concern [10]. Similarly, the requirements on elemental impurities in European Pharmacopoeia 11.2 (Ph. Eur.) are aligned with the ICH Q3D(R2) guideline [11]. Evaluating the total concentration of elemental impurities in drugs requires accurate extraction procedures and precise analytical methods. As the majority of all drugs contain both API and excipients, the final dosage form constitutes a complex sample matrix for total trace elemental quantification. Common for both the USP and the Ph. Eur. is the option of separate determination of trace elemental impurities in each substance used for drug production and summarizing the total amounts of trace elemental impurities to the final drug product [4,12]. Additionally, the drug manufacturer must ensure that the manufacturing, production, and packaging processes do not contribute to the total amount of trace elemental impurities [10].

Sample preparation is often the most critical step and is interlinked with successfully determining trace elemental impurities. Compared to the analysis of the component substances, analysis of the final dosage increases the sample complexity, and one must carefully evaluate the sample preparation strategy. Wet digestion, direct dissolution, and combustion are popular sample preparation procedures, followed by trace elemental quantification using inductively coupled plasma (ICP) spectrometry methods providing low limits of detection (LOD) [1,2,[13], [14], [15]]. Recent studies focus on the application of microwave-assisted acid digestion for sample preparation to evaluate trace elemental impurities in solid pharmaceuticals. The use of concentrated nitric acid, aqua regia, or inverse aqua regia are frequent acid choices. Pinheiro et al. (2019) evaluated microwave-assisted acid digestion of tablets using dilute or concentrated nitric acid solutions. The study found higher amounts of residual carbon in digests prepared using dilute nitric acids, indicating less complete digestion of organic molecules in the samples. However, the residual organic carbon did not affect the ICP analysis as internal standardization and aerosol dilution were used to correct matrix effects [14]. Few studies have alternatively used laser ablation ICP spectrometry as a preparation strategy for the direct determination of trace elemental analysis in solid API or drug formulations [[16], [17], [18], [19]], but the method has yet to be widely adopted for pharmaceuticals.

Ibuprofen drug products were chosen as the model substance for this study. Ibuprofen as API is synthesized through either of the two known reactions: the Boots or the Boots-Hoechst-Celanese reaction [20]. As these reactions use different elemental catalysts, each reaction potentially leaves a different trace elemental profile in the final drug product. The excipients used in the production of ibuprofen tablets are well-described and contain both organic and inorganic excipients [21,22], also imprinting different trace elemental signatures. In addition, ibuprofen as a model drug has the advantage of being commercially available from a wide range of manufacturers and with different formulations. Ibuprofen tablets are hence a useful model system to investigate the variation in elemental impurities among the different brands available on the European market.

As declared on the drug labels, all investigated ibuprofen drug products should contain Mg and/or Ti-containing excipients, these trace elements were therefore included for analysis. Fe, Zn, and Mn are, by the ICH Q3D(R1) guideline, described as trace elements to consider for analysis as these elements are possible contaminants from drug production processes. Common excipients (and some APIs) do intentionally contain Fe and Zn, making these elements useful for trace elemental pattern analysis. Thus, Mg, Ti, Fe, and Zn were intentionally included for analysis in this study. The elements Li, Sn, Os, Hg, and Tl described in the ICH Q3D(R1) guideline were not included in this study primarily due to analytical challenges associated with both sample preparation and limitations of ICP mass spectrometry i.e., Os requires stabilization of the sample solution to avoid the formation of volatile OsO_4_ [23], Hg demonstrates challenges with carry-over effect during ICP-MS analysis [24], Li has a high and varying background in ICP-MS [25], Sn potentially vaporizes as SnCl_4_ and/or forms colloidal precipitation in the presence of hydrochloric acid (HCl) [26]. Previous studies describe methods for measuring all 24 ICH Q3D elements including Li, Sn, Os, Hg, and Tl [1,7,14] for toxicological evaluation of pharmaceuticals.

The aim of this study was to establish an ICP-MS-based multi trace elemental analysis method (Mg, Ti, V, Cr, Mn, Cu, Fe, Co, Ni, Zn, As, Se, Mo, Ru, Rh, Pd, Ag, Cd, Sb, Ba, Ir, Pt, Au, and Pb) using small sample masses and digestion volumes for solid pharmaceuticals in relation to the ICH Q3D(R1) guideline as well as to investigate the possibility of linking trace element content to tablet drug formulation, and to distinguish between branded and generic drug products from the trace elemental profile.

## 2 Materials and methods

### 2.1 Samples

Ten ibuprofen drug products were purchased over the counter in Denmark, Sweden, and Spain (Table 1).

**Table 1:** Ibuprofen drug product information.

Drug product	Dosis	Country of purchase
Ibu1^a^	400 mg	Denmark
Ibu2	400 mg	Denmark
Ibu3	400 mg	Denmark
Ibu4	400 mg	Denmark
Ibu5	400 mg	Denmark
Ibu6^a^	400 mg	Sweden
Ibu7	400 mg	Sweden
Ibu8	400 mg	Sweden
Ibu9	400 mg	Sweden
Ibu10	400 mg	Spain

a Ibu1 and Ibu6 were produced by the same manufacturer but purchased in different countries.

### 2.2 In-house quality control material

An in-house quality control material was prepared to evaluate the interday method repeatability. 60 ibuprofen 400 mg tablets (purchased in Denmark), with an average tablet weight of 567.7 mg, were ground using a porcelain mortar and pestle (Haldenwanger, Berlin, Germany). The ground powder was sieved through a stainless steel 600 μm Mesh sieve (Retsch, Haan, Germany) to ensure homogeneous particle size.

### 2.3 Sample preparation

Three tablets from each product were ground and homogenized using a porcelain mortar and pestle. 10 mg tablet powder or in-house quality control material was weighed using an analytical balance (Mettler Toledo, Columbus, OH, USA) and transferred directly into 5 mL glass microwave digestion vials (Anton Paar GmbH, Graz, Austria). 525 μL concentrated 65% nitric acid (HNO_3_) (EMSURE, Merck KGaA, Darmstadt, Germany) and 175 μL concentrated 37% HCl (EMSURE, Merck KGaA, Darmstadt, Germany) were added to each microwave digestion vial. The vials were gently whirled by hand and allowed to pre-digest for 5 min. Following, 200 μL concentrated 30% H_2_O_2_ (EMSURE, Merck KGaA, Darmstadt, Germany) was added and pre-digested for 15 min. The digestion vials were sealed (Teflon seal) and capped (polycarbonate) by hand (pressure limit 20 Bar) and digested using a Synthos 3000 microwave (Anton Paar GmbH, Graz, Austria) equipped with a 64-position carousel. All tablet products were digested in triplicates. Procedural blank samples were prepared as described above without tablet powder. The digestion program is shown in Table 2.

**Table 2:** Microwave operational parameters used during acid digestion of ibuprofen drug products.

Step	Procedure	Time [min]	Power [W]	Temperature [°C]
1	Ramp	5	0–100	160
2	Hold	5	100	160
3	Ramp	10	100–200	160
4	Hold	10	200	160
5	Cooling	10	0	0

**Fig. 1:**
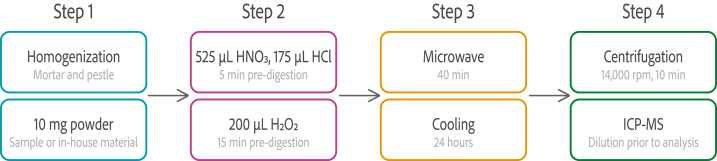
Flow chart of the digestion process.

After the microwave procedure, the vials cooled for additional 24 h in a fume hood for the convenience of the following sample analysis. However, the sample can be used after reaching room temperature. The digests were transferred to 1.5 mL polypropylene micro tubes (Th. Geyer GmbH, Renningen, Germany) and centrifuged for 10 min at 14,000 rpm (Minispin Plus, Eppendorf, Hamburg, Germany). All tablet digests contained residual solids. Before ICP-MS analysis, 600 μL of the digested tablet sample solutions were transferred to a 15 mL polypropylene autosampler tubes (VWR, Radnor, PA, USA), 50 μL internal standard solution (5.00 μg/L Y and In) was added, and ultrapure water (>18,2 M**Ω** cm, Merck KGaA, Darmstadt, Germany) was added to a final volume of 10.00 mL. The same procedure was applied for blank digestions. For the detection of samples with high concentrations of Mg, dilutions were performed separately to fit the linear calibration range. 45.0 μL of the digested tablet solution and 50.0 μL internal standard solution were transferred to a 15 mL polypropylene autosampler tube and diluted to 10.00 mL with ultrapure water. For measurement of Mg in the in-house quality control material, 90.0 μL of the digested tablet solution and 50.0 μL internal standard solution were transferred to a 15 mL polypropylene autosampler tube and diluted to 10.00 mL with ultrapure water. Spiked samples were prepared as described below. The diluted solutions were analyzed using ICP-MS. An overview of the total sample preparation procedure is presented in Fig. 1.

### 2.4 Internal standard preparation

The internal standard solution was prepared using 1000 mg/L Y and In single element ICP-MS calibration standards (PerkinElmer, Waltham, MA, USA) diluted to 1000 μg/L with 2% HNO_3_ (EMSURE, Merck KGaA, Darmstadt, Germany). The 2% HNO_3_ diluted internal standard solution was manually added to all blank, standard, and sample solutions to a concentration of 5.00 μg/L before ICP-MS analysis.

### 2.5 Calibration standards

Throughout this study, two ICP-MS multi-elemental solutions were used for the preparation of calibration standards: Standard stock A contained 100 mg/L (Ag, Al, As, B, Ba, Be, Ca, Cd, Co, Cr, Cu, Fe, K, Mg, Mn, Mo, Na, Ni, Pb, Sb, Se, Si, Ti, Tl, V and Zn) (SPC Science, Quebec, Canada) and standard stock B contained 10 mg/L (Au, Ir, Pd, Pt, Os, Rh, and Ru) (CPI International, Santa Rosa, CA, USA). A freshly prepared acid mix of 0.1% HCl (37% fuming, EMSURE, Merck KGaA, Darmstadt, Germany) and 0.65% HNO_3_ (EMSURE, Merck KGaA, Darmstadt, Germany) prepared in ultrapure water was used for dilution of the calibration standards. Calibration standards were prepared daily in the concentration ranges of 0–20.00 μg/L for standard A and 0–2.00 μg/L for standard B. To all calibration standards, 50.00 μL 1000 μg/L Y and In was added, and the calibration standards were diluted with the acid mixture to a final volume of 10.00 mL (5.00 μg/L internal standard). A 7-point standard curve was applied for both calibration standards.

### 2.6 Preparation of spiked samples

Three-level spike recovery was evaluated in three different ibuprofen samples (Ibu4, Ibu5, and in-house quality control material). The spike levels 2.50 μg/L, 5.00 μg/L, and 7.50 μg/L were chosen to primarily match the Mg, Ti, and Fe levels, roughly corresponding to spike levels of 0.5, 1, and 1.5 times the concentration in the sample solution. 150 μL of the digested tablet solution was transferred to four 15 mL polypropylene autosampler tubes. For spike recovery detection of Mg, only 90.0 μL of the digested tablet solution was used. The first subsample was left unspiked. The remaining three were added 6.25 μL, 12.50 μL, and 18.75 μL of 1000 μg/L multi-element standard A or B, respectively. 12.5 μL internal standard solution (5.0 μg/L final concentration) was added to unspiked and spiked samples and diluted with ultrapure water to a final total volume of 2.50 mL before ICP-MS analysis.

### 2.7 Instrumentation

The trace elemental concentrations were quantified using an Agilent Technologies 8800 Triple Quadrupole ICP-MS (Agilent Technologies, Santa Clara, CA, USA) equipped with an Agilent SPS4 autosampler. The ICP-MS was mounted with a cooled (5 °C) Scott-type spray chamber (Agilent Technologies), a SeaSpray U-series concentric nebulizer (Glass Expansion, Weilburg, Germany), nickel sampler and skimmer cones (Agilent Technologies, Santa Clara, CA, USA). The instrument was located at University of Copenhagen, Denmark. Table 3 describes the ICP-MS operating parameters used throughout the analyses. Auto optimization was performed daily.

**Table 3:** Agilent Technologies 8800 Triple Quadrupole operating conditions during the analysis of trace elemental impurities in ibuprofen drug products.

Parameter	Value
RF applied power [W]^#^	1550
Plasma gas flow rate [L/min]	15.0
Auxiliary gas flow rate [L/min]	0.90
Nebulizer gas flow rate [L/min]^#^	1.21
Collision gas (He) flow rate [mL/min]	4.5
Lens voltage [V]^#^	−130
Selected isotopes [*m*/*z*]	^24^Mg,^47^Ti,^51^V,^52^Cr,^55^Mn,^56^Fe,^59^Co,^60^Ni,^63^Cu,^66^Zn,^75^As,^82^Se,^98^Mo,^101^Ru,^103^Rh,^105^Pd,^107^Ag,^111^Cd,^121^Sb,^137^Ba,^93^Ir,^195^Pt,^197^Au,^208^Pb
Internal standards [*m*/*z*]	^89^Y,^115^In
Analysis time pr. solution [s]	65
Wash [s]	60
Sweeps/replicate	10
Number of replicates	5
Dwell time [ms]	300
Sample uptake rate [mL/min]	0.3
Integration time [s]	0.3

# Optimized daily.

### 2.8 Method validation

#### 2.8.1 Linearity of standard calibration curves

The linearity was assessed using ICP-MS-certified calibration standards. The ratio of counts per second for the trace element isotopes of interest and the internal standard isotope was plotted against the standard concentration using linear regression. The linearity was evaluated according to R^2^-values (≥0.9990) and the visual assessment of the fitted plot.

#### 2.8.2 Limit of detection

The LOD of trace elements in the ibuprofen tablet products was determined using Equation 1

(1) $$LOD=3.3\cdot \frac{\sigma }{S}\cdot DF$$

where σ is the standard deviation of the measured counts per second of ten blank samples, S is the slope of the calibration curve (μg/g·cps^−1^). The LOD was calculated with and without the total dilution factor (DF) to present LOD in the tablet powder and in the sample solution measured on the ICP-MS instrument, respectively.

#### 2.8.3 Accuracy and precision

The method accuracy was evaluated as the percentage spike recovery described in Equation 2

(2) $${Recovery}_{\%}=\frac{{conc}_{spiked\;sample}-{conc}_{sample}}{{conc}_{spike}}\cdot 100\%$$

where conc_spike sample_ is the measured concentration of the spiked sample, conc_sample_ is the measured concentration of the sample, and conc_spike_ is the theoretical added spike concentration. The acceptance criteria for spike recovery are 70–150%, which is in accordance with the USP *<233> Elemental Impurities - Procedures* and Ph. Eur. 2.4.20. *Determination of elemental impurities* monographs [12], as no acceptance criteria are specified in the ICH Q3D(R2) guideline for method validation [5].

The method precision was assessed according to the interday repeatability of the in-house quality control material and reported as the average measured concentration and the standard deviation. Duplicates of the in-house quality control materials were digested and diluted as the samples described in section 2.3 and analyzed on each day of ICP-MS analysis.

### 2.9 Principal component analysis

Exploratory data analysis was performed on the measured trace elemental concentrations in ibuprofen drug products using MATLAB R2022b in combination with PLS Toolbox 9.2 for MATLAB (Eigenvector Research Inc., Manson, WA, USA). For each measured trace element, the measured trace elemental concentration was standardized (standard normal variate) using Equation 3

(3) $${TE}_{standardized}=\frac{{TE}_{measured}-{TE}_{all,avg}}{{TE}_{all,stdev}}$$

where ${TE}_{standardized}$ is the standardized value of the measured trace elemental concentration, ${TE}_{all,avg}$ is the average of all measured trace elemental concentrations for each trace element, and ${TE}_{all,stdev}$ is the standard deviation of all measured trace elemental concentrations for each trace element. Trace elemental concentrations below LOD were set to zero for the PCA analysis. The root mean square error of validation (RMSEV) versus root mean square error of cross-validation (RMSECV) was plotted as a function of principal components (PCs) to determine the number of PCs. The Hotelling's T^2^ Q-residuals plot was used for outlier determination.

### 2.10 Software

Agilent MassHunter version 4.5 and Microsoft Excel version 16.72 were used for ICP-MS data preliminary evaluation and extraction. MATLAB R2022b and PLS Toolbox 9.2 for MATLAB were used for data evaluation and exploratory data analysis. All graphical illustrations were made using MATLAB R2022b and Adobe Illustrator 2023.

## 3 Results and discussion

### 3.1 Digestion procedure

Closed vessel digestion was chosen to minimize the loss of volatile trace elemental impurities and to reduce the residual carbon content [14,27]. Prior to the final digestion procedure (see section 2.3.), digestion optimization was performed to reduce the amount of residual solids, leaking, and exploding vials. The optimization parameters included varying the amount of sample (10–20 mg), the microwave digestion temperature (160–200 °C), the microwave power (200–400 W), and the digestion time (40–70 min). Despite optimization, residual solid was always observed, and the color of the digest ranged from slightly yellow to clear (data not shown). Therefore, a centrifugation step was integrated into the sample preparation procedure to sediment and separate insoluble residuals of ibuprofen. ICP-MS sample solutions were visually inspected for remaining particles before analysis. To prevent potential clogging and improve sensitivity, a SeaSpray concentric nebulizer was mounted as the nebulizer tolerates up to 20% salt solutions and 75 μm particles [28].

According to the drug labels, all ibuprofen drug products evaluated in this study contained insoluble or partially insoluble excipients such as SiO_2_ and TiO_2_ [21,22]. The composition of residual solids was not determined, as this was beyond the scope of this study. However, based on the list of excipients present in the ibuprofen drug products, we suggest the residual solids contained primarily silicon dioxide, as titanium dioxide is soluble in hot concentrated HNO_3_ and HCl [29]. Analytes can be trapped in residual solids, and one must carefully evaluate alternative digestion acids, temperatures, etc., to improve the digestion process before introducing a centrifugation procedure. However, for drugs containing insoluble or partially insoluble excipients such as silicon dioxide and titanium dioxide, the digestion possibilities are limited. Digestion of ibuprofen using hydrofluoric acid has been attempted and failed, as white and flaky insoluble residuals were formed (data not shown). The use of hydrofluoric acid has likely formed insoluble fluorides [30] and was discontinued. A study from Pinheiro et al. (2019) also reports residual solids after the digestion of nine solid tablet products remedied by centrifugation. The authors did not further discuss the consequence of removing residual solids but did demonstrate great spike recoveries after closed vessel digestion with HNO_3_ [14]. de Mello et al. (2022) investigated partial digestion of six inorganic sample matrices using dilute solutions of HNO_3_ and concentrated H_2_O_2_ for closed vessel microwave-assisted acid digestion. Sample digestion using concentrated HNO_3_ and HF was used as a reference for full digestion. A total of 15 elements (Na, Mg, P, S, Al, Si, K, Ca, Cr, Mn, Fe, Ni, Cu, Zn, and Pb) were analyzed by ICP-OES. The authors did not report any residual solids after partial digestion of the studied inorganic compounds but concluded that despite various inorganic sample matrices, acceptable recoveries (80–120%) were achieved for most elements by partial digestion [31].

The interior design of the microwave also puts limitations on the digestion procedure. The tablet powder sample amount is limited by the size of the digestion vials setting high standards for the homogenization of the tablet powder to reduce sample variation. Throughout this study, the digestion temperature was kept constant at 160 °C, as higher temperatures resulted in leaking or exploding digestion vials. The variety of API and excipients constituting the drug are almost infinite but are crucial for the digestion procedure, and critical digestion parameters (e.g., acid composition, digestion temperature, microwave equipment, etc.) should be consolidated on a case-by-case based decision.

### 3.2 Trace elements in ibuprofen drug products

The trace elemental composition of the ten ibuprofen drug products is described in Table 4. Only Mg, Ti, Cr, Mn, Fe, and Ni were determined in the ibuprofen products, and not all elements were detected in each product. For V, Cu, Co, Zn, As, Se, Mo, Ru, Rh, Pd, Ag, Cd, Sb, Ba, Ir, Pt, Au, and Pb, no concentrations above LOD were determined in any of the products (acquisition mode, spike recoveries, and LODs are found in supplementary materials Table S1).

**Table 4:** The measured trace elemental concentrations in ibuprofen drug products presented as the average concentration of three replicates in μg element per gram tablet powder ± the standard deviation. The limit of detection was measured in the sample solution (μg/L) and back-calculated to the tablet powder (μg/g) using ten replicates. Only concentrations above the limit of detection (LOD) are presented. The acquisition mode is reported as the measured isotope, the internal standard isotope, and the ICP-MS collision gas mode.

Element (n=3)	Acquisition mode	Ibu1	Ibu2	Ibu3	Ibu4	Ibu5	Ibu6	Ibu7	Ibu8	Ibu9	Ibu10	Limit of detection in the sample solution [μg/L] and in the tablet powder [μg/g] (n=10)
Mg	^24^Mg/^89^Y, No gas	357.7 ± 19.0	468.3 ± 9.1	<LOD	468.7 ± 24.6	1119.4 ± 31.6	358.8 ± 24.3	478.5 ± 29.6	442.0 ± 24.8	399.8 ± 8.9	218.7 ± 22.8	3.1 μg/L, 45.9 μg/g
Ti	^47^Ti/^89^Y, No gas	17.1 ± 4.6	10.8 ± 14.6	10.4 ± 3.7	46.6 ± 12.4	1.2 ± 0.3	13.8 ± 4.4	15.7 ± 5.7	3.7 ± 1.0	4.3 ± 7.0	<LOD	1.3 μg/L, 2.0 μg/g
Cr	^52^Cr/^89^Y, He gas	<LOD	<LOD	<LOD	1.0 ± 1.4	3.4 ± 5.3	<LOD	<LOD	<LOD	<LOD	<LOD	0.2 μg/L, 0.4 μg/g
Mn	^55^Mn/^89^Y, No gas	<LOD	0.68 ± 0.03	<LOD	<LOD	<LOD	0.14 ± 0.13	<LOD	<LOD	<LOD	<LOD	0.06 μg/L, 0.09 μg/g
Fe	^56^Fe/^89^Y, He gas	<LOD	<LOD	<LOD	9.9 ± 5.3	61.7 ± 22.7	<LOD	<LOD	<LOD	<LOD	<LOD	4.5 μg/L, 6.8 μg/g
Ni	^60^Ni/^89^Y, He gas	<LOD	<LOD	0.12 ± 0.14	<LOD	1.6 ± 2.5	<LOD	<LOD	<LOD	<LOD	<LOD	0.06 μg/L, 0.09 μg/g

High concentrations of Mg were found for all products other than Ibu3 where no Mg was reported above LOD. The presence of high Mg concentrations is likely interlinked with using Mg-stearate as a tablet glidant excipient [32]. All ibuprofen products that contained Mg have Mg-stearate listed as an excipient [21,22]. Ibu3 did not have Mg-stearate listed as an excipient but contained stearic acid, a common glidant substitute for Mg-stearate [32], which explains why Mg was not found in this product. Ti was determined in all the ibuprofen products except Ibu10. Ti in the form of TiO_2_ is widely used as a white pigment in tablet production [32] and was present on the lists of ingredients for all drug products except Ibu10 [21,22].

Ibu1 and Ibu6 were produced by the same manufacturer but were purchased in Denmark and Sweden, respectively. Presumably Ibu1 and Ibu6 have been manufactured at the same production plant and demonstrated similar concentrations of Mg (*t*-test two-tailed, p-value: 0.955) and Ti (*t*-test two-tailed, p-value 0.419). Mn was only found in the Ibu6, though the Mn concentration was low and might not have been detected in Ibu1 due to sample inhomogeneity. Apart from Ibu6, only Ibu2 contained a low concentration of Mn.

Cr and Fe were only determined for Ibu4 and Ibu5, where Ibu5 showed the highest concentrations of both elements. Ni was found in both Ibu4 and Ibu5. A study by Li et al. (2015) investigated the trace elemental impurities of pharmaceutical excipients. The author studied 24 trace elements (Li, V, Cr, Co, Ni, Cu, As, Se, Mo, Ru, Rh, Pd, Ag, Cd, Sn, Sb, Ba, Os, Ir, Pt, Au, Hg, Tl, and Pb) in 31 common excipients [22,33]. The study demonstrated that trace elemental impurities like Cr and Ni are found in excipients such as TiO_2_, SiO_2_, Mg-Stearate, and hypromellose, excipients listed in Ibu3, Ibu4, and Ibu5 [22]. Another possible source of Fe, Ni, and Cr contamination is from the steel/metal equipment frequently used for tablet drug production. However, considering the measured trace elements in the ten ibuprofen drug products, none of the products contained trace elements at concentrations of concern or exceeded the permitted daily exposure limits described by the ICH Q3D(R1) guideline [4].

### 3.3 Principal component analysis of ibuprofen

**Fig. 2:**
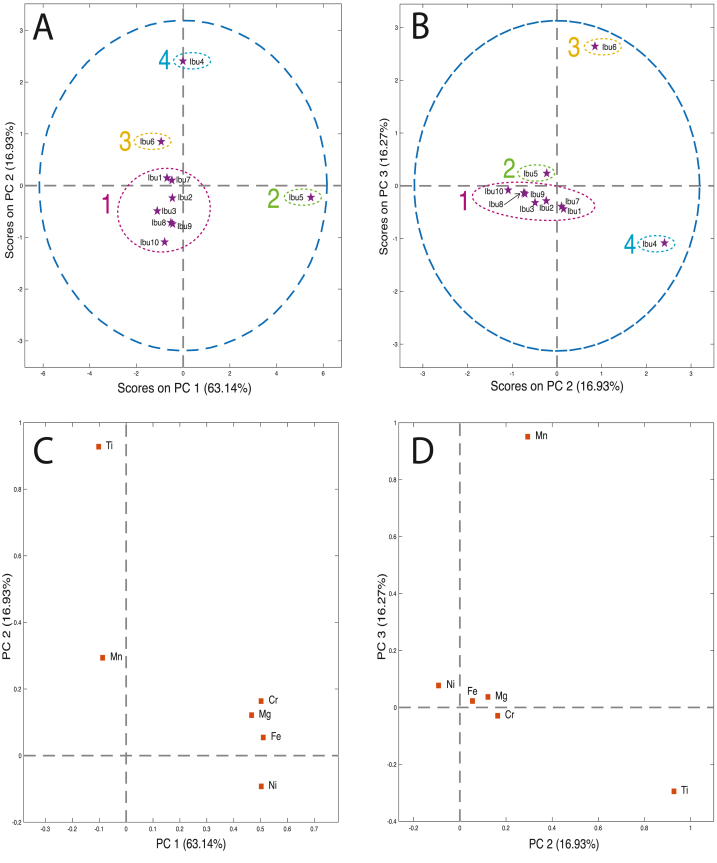
Principal component analysis of ten ibuprofen drug products. **A)** Score plot of PC1/PC2 () and **B)** Score plot of PC2/PC3 () formed four groups depicted as 1 (magenta), 2 (green), 3 (yellow), and 4 (blue). **C)** Loading plot of PC1/PC2 (), and **D)** Loading plot of PC2/PC3 ().

PCA was used to investigate possible trace element patterns among the ten ibuprofen products. The trace elements presented in Table 4 were standardized prior to analysis. From Hotelling's T^2^ Q-residuals plot, no outliers were determined, and three PCs were chosen based on the RMSEV/RMSECV versus PC plots, describing 96% of the data variation (data not shown). PC1 and PC2 accounted for 63% and 17% of the variance, respectively. The score and loading pots of PC1/PC2 and PC2/PC3 are depicted in Fig. 2.

PCA was useful in distinguishing between the ibuprofen drug products and addressing the variance of the trace elemental data. As seen on the score plots, the ibuprofen drug products were clearly separated and roughly formed four groups, including *(1)* the ibuprofen products located around the origin (Ibu1, Ibu2, Ibu3, Ibu7, Ibu8, Ibu9, and Ibu10), *(2)* Ibu5, *(3)* Ibu6, and *(4)* Ibu4 (Fig. 2A and B). Ibu8 and Ibu9 were closely located on the two score plots (Fig. 2A and B), indicating that their trace elemental profiles were comparable across the variance found in the three PCs. In addition, Ibu1 and Ibu7 were comparable across the variance captured by PC2 and PC3 (Fig. 2B). Examining the loading plot of PC1/PC2, PC1 was correlated with Mg, Fe, and Cr, whereas PC2 was primarily correlated with Ti and Mn (Fig. 2C). Ibu5 showed a positive correlation for PC1 associated with the presence of Mg, Fe, and Cr, whereas Ibu4 was correlated with PC2 and high concentrations of Ti. The origin-centered points *(1)* had smaller variations in Mg, Fe, Cr, and Ti concentrations but variating enough to be clearly separated on the score plot (Fig. 2A). To some extent, PC2 was also correlated with Mn, as seen in Figure 2A-D, explaining why Ibu1 was not clustered with Ibu6.

### 3.4 Method validation

#### 3.4.1 Linearity

The calibration standard solutions were prepared separately from standard stock solution A and solution B and were not mixed to avoid potential ICP-MS interferences and precipitation. All 24 trace elements demonstrated linearity in the investigated range of 0–20.00 μg/L (standard stock solution A) and 0–2.00 μg/L (standard stock solution B). The R^2^-values were ≥0.9991 for all 24 trace elements.

#### 3.4.2 Limit of detection

LOD was reported for both the ICP-MS measured sample solution (μg/L) and back-calculated to the tablet powder (μg/g) (Table 4). Gu et al. (2021) presented LOD values of 0.09 μg/L for Cr and 0.01 μg/L for Ni in 2% HNO3-1% HCl solutions, whereas Pinheiro et al. (2019) demonstrated LOQ values for Cr and Ni of 0.23 μg/g and 0.32 μg/g (equivalent to LODs of 0.076 μg/g and 0.10 μg/g, respectively) in dilute HNO_3_–HCl solutions [14]. Both Gu et al. (2021) and Pinheiro et al. (2019) used ICP-MS for analysis, and the LOD values reported in this study were in agreement with the literature values. Van Hoecke et al. (2012) presented comparable LOD values for Cr and Ni and additionally reported on Mn and Fe LOD values of 0.1 μg/L and 1 μg/L, respectively, in dilute HCl–HNO_3_ solutions [34]. Compared to Van Hoecke et al. (2012), Fe demonstrated a higher LOD in this study, however, the measured Fe concentrations were significantly higher than the LOD. The LOD values from this study were in good agreement with previously reported values and were found satisfactory for the quantitative determination of trace elements in ibuprofen (see also supplementary materials Table S1 for other elements). To the best of our knowledge, Mg and Ti have not previously been measured in solid pharmaceuticals. However, the LOD values for Mg and Ti were relatively low, and the measured Mg and Ti concentrations were significantly higher than the LOD.

#### 3.4.3 Method accuracy and precision

The method accuracy was evaluated through spike recoveries in three different ibuprofen sample matrices. The recovery percentages and relative standard deviations are found in Table 5. Ti, Cr, Mn, Fe, and Ni demonstrated recoveries ranging from 94 to 119% across all spiked ibuprofen matrices, which falls within the USP and Ph. Eur. recovery acceptance criteria. Unsatisfactory Mg recoveries were found for the lowest spike concentration. In general, too high recoveries of Mg were detected in the Ibu5 spike matrix. As Mg was measured ^24^Mg isotope in no collision gas mode, one must also consider the potential effect of residual carbon as polyatomic interference (^12^C_2_^+^) affecting and leading to high spike recoveries [35]. The Ibu5 sample contained the highest Mg concentrations (Table 4), and additional dilution of the Ibu5 spike matrix could potentially provide better Mg spike recoveries. Acceptable Mg spike recoveries of 75–134% were reported for mid and high spike levels (5.0 μg/L and 7.5 μg/L, respectively) in Ibu4 and the in-house quality control material.

**Table 5:** Recovery percentages and relative standard deviations (%) for three spiked ibuprofen drug products (Ibu4, Ibu5, and in-house quality control material) quantified by ICP-MS (n = 2). Spike levels (2.5 μg/L, 5.0 μg/L, and 7.5 μg/L) were chosen to match the Mg, Ti, and Fe concentrations in the measured sample solution.

	Ibu4			Ibu5			In-house quality control material		
Spike level	2.5 μg/L (50%)	5.0 μg/L (100%)	7.5 μg/L (150%)	2.5 μg/L (50%)	5.0 μg/L (100%)	7.5 μg/L (150%)	2.5 μg/L (50%)	5.0 μg/L (100%)	7.5 μg/L (150%)
Mg†	30	92	75	223	118	193	155	87	134
Ti	115.2 (7.2)	107.9 (1.0)	94.0 (4.8)	108.2 (3.8)	105.2 (1.8)	106.2 (0.3)	106.7 (8.8)	112.8 (1.8)	113.8 (0.7)
Cr	117.5 (1.5)	112.7 (0.1)	101.2 (14.5)	112.9 (4.3)	110.6 (0.2)	111.1 (1.0)	104.2 (5.4)	109.4 (2.6)	112.0 (1.4)
Mn	117.9 (0.9)	111.8 (2.1)	99.5 (13.6)	113.0 (2.2)	108.6 (1.3)	107.9 (0.9)	104.4 (1.4)	105.1 (0.1)	109.8 (3.6)
Fe	<LOD	114.5 (12.0)	96.1 (18.6)	<LOD	95.8 (9.4)	97.3 (0.1)	<LOD	117.1 (5.3)	114.0 (19.6)
Ni	119.3 (0.3)	115.1 (0.7)	103.3 (14.4)	117.1 (2.2)	113.1 (1.1)	113.1 (0.5)	108.0 (2.1)	112.1 (1.3)	113.6 (3.8)

† Single determination on spike recovery due to additional separate dilution for Mg measurements, no standard deviations are reported.

The method precision was evaluated through the trace elemental concentration of Mg. The in-house quality control material has magnesium stearate declared as an excipient, and detectable concentrations of Mg were therefore expected. The analyses of the ibuprofen drug products were performed over five days, and the in-house quality control material was included for each analysis in total 15 individual measurements from microwave digestion to ICP-MS detection were performed. The average concentration of Mg was 268.1 ± 22.1 μg/g with a 95% confidence interval of [258.1 μg/g; 278.1 μg/g] and a relative standard deviation of 8.2%. The in-house quality control material has passed a comparable homogenization and sample preparation procedure as the sample but demonstrated a higher relative standard deviation in comparison to most spike recoveries found in Table 5. However, a higher relative standard deviation would be expected for the in-house quality control material, as this material has passed the entire sample preparation procedure, therefore, having multiple steps contributing to the variation compared to spiked samples. The constant determination of the Mg across multiple days of analysis points towards that the sample preparation procedure was reproducible across days. The in-house quality control material was tested in a different laboratory for comparison following the same sample preparation and digestion procedure described in section 2.3. Here, three individual digestions and ICP-MS analyses were performed, and the average Mg concentration of the in-house quality control material was 270.8 ± 12.2 μg/g. No significant differences in the average Mg concentration of the in-house quality control material (*t*-test two-tailed, p-value 0.851) were found across the two laboratories.

A relative standard deviation better than 8% might be possible to obtain with a more homogeneous quality control material or by using a certified reference material. However, no certified National Institute of Standards and Technology (NIST) or European Commission's Joint Research Centre reference material exists for elemental impurities in pharmaceuticals [36,37]. NIST has “*multielement tablets*” (SRM 3294) available, but this reference material is intended for food and agricultural analyses [36]. A good reference material must reflect the sample matrix of interest, and the NIST SRM 3294 was considered insufficient as the sample matrix differs from a pharmaceutical tablet formulation, and the certified trace elemental concentrations were unnaturally high compared to trace elemental impurities found in pharmaceutical tablets. In addition, the NIST SRM 3294 has not been produced under comparable pharmaceutical manufacturing conditions and was therefore not considered as reference material for this study. Reference materials are crucial to obtaining high-quality methods with good precision and accuracy, and the in-house quality control material was used as the currently best alternative to a certified reference material.

## 4 Conclusion

The aim of this study was to establish an ICP-MS based multi trace elemental analysis method for solid pharmaceuticals in relation to the ICH Q3D(R1) guideline to investigate the possibility of linking trace element content to tablet drug formulation and to distinguish between drug products from the trace elemental profile. The method was established by evaluating the trace elemental composition of ten European ibuprofen products using only 10 mg drug product and 900 μL acid. Of the 24 trace elements in ibuprofen drug products, only Mg, Fe, Ti, Mn, Cr and Ni were above the LOD of the current study, and none exceeded the ICH Q3D(R1) permitted daily exposure limits. The European ibuprofen products were considered safe, with no trace elemental-related toxicological risks in the use of drug products. Combining the trace elemental profiles with PCA, it was possible to group the different ibuprofen products and relate the trace elemental remnants to drug formulation and production methods.

Due to the lack of certified reference material, the ICP-MS method was validated through self-produced in-house quality control material and spike recoveries. Great spike recoveries were obtained for most trace elements. Despite multiple dilutions, Mg showed poor spike recoveries for low spike concentrations when the spike matrix contained high Mg concentrations. The experiments and analyses were not conducted in clean-room facilities, potentially affecting the LODs. Lowering the LODs would eventually provide more reliable data for evaluation and PCA. Separating residual solids after microwave-assisted acid digestion was needed as the ibuprofen drug products contained insoluble SiO_2_. Improved digestion methods (i.e., pressure control and higher temperatures) are required to obtain clear digestion solutions without residual solids for drugs such as ibuprofen.

In summary, the presented method is applicable to evaluate trace elemental profiles (e.g., Mg, Ti, V, Cr, Mn, Cu, Fe, Co, Ni, Zn, As, Se, Mo, Ru, Rh, Pd, Ag, Cd, Sb, Ba, Ir, Pt, Au, and Pb) of solid drugs comparable to ibuprofen, depending on compositions and individual laboratory detection limits.

## Data availability statement

The data has not been submitted to any public repository. The data is available upon request to holmfred@stanford.edu or stefan.sturup@sund.ku.dk.

## CRediT authorship contribution statement

**Else Holmfred:** Conceptualization, Data curation, Formal analysis, Funding acquisition, Investigation, Methodology, Project administration, Resources, Software, Supervision, Visualization, Writing – original draft, Writing – review & editing. **Abdulla Alrijjal:** Data curation, Formal analysis, Investigation, Methodology. **C. Page Chamberlain:** Resources, Supervision, Writing – review & editing. **Katharine Maher:** Resources, Supervision, Writing – review & editing. **Stefan Stürup:** Conceptualization, Funding acquisition, Methodology, Project administration, Resources, Supervision, Writing – original draft, Writing – review & editing.

## Declaration of competing interest

The authors declare that they have no known competing financial interests or personal relationships that could have appeared to influence the work reported in this paper.
