# Supplementary material for: Determination of trace elements in ibuprofen drug products using microwave-assisted acid digestion and inductively coupled plasma-mass spectrometry
Source: Heliyon. 2023 Dec 10;10(1):e23566. doi: 10.1016/j.heliyon.2023.e23566 (PMC10776936; doi:10.1016/j.heliyon.2023.e23566)
Supplement: Multimedia component 1 [file mmc1.docx]

Supplementary information

**Table S1.** No concentrations above the limit of detection were determined for V, Cu, Co, Zn, As, Se, Mo, Ru, Rh, Pd, Ag, Cd, Sb, Ba, Ir, Pt, Au, and Pb. Percentage recoveries and relative standard deviation (%), the limit of detection in the sample solution (µg/L) and tablet powder (µg/g), as well as the ICP-MS acquisition mode (n=2), are found below.

|  | Ibu4 | | | Ibu5 | | | In-house quality control material | | | Limit of detection in the sample solution [µg/L] and in the tablet powder  [µg/g] | Acquisition mode | |
| --- | --- | --- | --- | --- | --- | --- | --- | --- | --- | --- | --- | --- |
| Spike level | 2.5 µg/L (50%) | 5.0 µg/L (100%) | 7.5 µg/L  (150%) | 2.5 µg/L (50%) | 5.0 µg/L (100%) | 7.5 µg/L  (150%) | 2.5 µg/L (50%) | 5.0 µg/L (100%) | 7.5 µg/L  (150%) |  |  |  |
| V | ND^#^ | ND^#^ | ND^#^ | ND^#^ | ND^#^ | ND^#^ | ND^#^ | ND^#^ | ND^#^ | 0.7 µg/L,  1 µg/g | ^51^V/^89^Y,  He gas |  |
| Cu | 109.8 (0.5) | 104.9 (1.1) | 94.6 (14.6) | 102.9 (3.1) | 100.8 (0.1) | 101.6 (0.1) | 96.8 (4.9) | 102.2 (2.9) | 106.1 (0.9) | 0.6 µg/L,  0.9 µg/g | ^63^Cu/^89^Y,  No gas |  |
| Co | 121.1 (0.1) | 115.5 (0.2) | 103.7 (14.8) | 117.3 (2.7) | 114.0 (1.0) | 113.4 (0.6) | 106.5 (2.1) | 112.1 (1.3) | 113.6 (3.8) | 0.07 µg/L,  0.1 µg/g | ^59^Co/^89^Y,  He gas |  |
| Zn | <LOD | 113.8 (3.4) | 103.1 (18.2) | <LOD | 110.9 (0.6) | 112.1 (2.5) | <LOD) | 108.8 (1.4) | 111.7 (1.9) | 4 µg/L,  6 µg/g | ^66^Zn/^89^Y,  He gas |  |
| As | 112.0 (1.1) | 111.4 (0.7) | 101.2 (15.0) | 105.6 (4.0) | 109.4 (0.0) | 108.4 (1.5) | 98.4 (3.5) | 104.7 (2.0) | 109.8 (0.7) | 0.03 µg/L,  0.04 µg/g | ^75^As/^89^Y,  He gas |  |
| Se | <LOD | 74.6 (1.5) | 67.7 (15.5) | <LOD | 70.9 (2.6) | 73.3 (2.0) | <LOD | 105.1 (2.2) | 108.5 (0.5) | 3 µg/L,  4 µg/g | ^82^Se/^89^Y,  No gas |  |
| Mo | 115.9 (0.3) | 109.1 (0.6) | 102.9 (19.1) | 106.9 (3.4) | 105.1 (0.2) | 105.9 (0.2) | 99.6 (5.1) | 105.0 (2.5) | 108.7 (0.9) | 0.1 µg/L,  0.2 µg/g | ^98^Mo/^89^Y,  No gas |  |
| Ru | 110.0 (2.0) | 104.9 (1.9) | 101.2 (2.7) | 112.4^&^ | 105.3^&^ | 106.5^&^ | 117.4 (5.5) | 107.5 (5.2) | 105.8 (5.9) | 0.002 µg/L,  0.003 µg/g | ^101^Ru/^89^Y,  No gas |  |
| Rh | 107.9 (2.3) | 107.5 (1.5) | 102.2 (3.8) | 111.2^&^ | 107.7^&^ | 110.4^&^ | 115.8 (5.5) | 109.5 (4.1) | 107.4 (6.4) | 0.03 µg/L,  0.04 µg/g | ^103^Ru/^115^In,  No gas |  |
| Pd | 98.2 (1.3) | 93.6 (2.1) | 90.8 (2.3) | 101.3^&^ | 95.7^&^ | 96.7^&^ | 105.2 (5.2) | 96.6 (5.8) | 95.3 (6.0) | 0.02 µg/L,  0.02 µg/g | ^105^Pd/^115^In,  No gas |  |
| Ag | 108.2 (1.4) | 103.7 (0.3) | 94.2 (14.1) | 101.2 (2.8) | 100.6 (0.4) | 101.7 (0.4) | 95.1 (7.0) | 101.3 (4.2) | 104.1 (1.0) | 0.02 µg/L,  0.03 µg/g | ^107^Ag/^115^In,  No gas |  |
| Cd | 95.9 (0.2) | 91.4 (0.3) | 83.3 (14.7) | 90.0 (2.9) | 96.3 (1.0) | 96.9 (0.4) | 83.6 (4.9) | 88.2 (2.5) | 91.3 (1.5) | 0.01 µg/L,  0.02 µg/g | ^111^Cd/^115^In,  No gas |  |
| Sb | 103.1 (0.1) | 98.5 (0.8) | 89.5 (14.6) | 97.9 (3.3) | 96.3 (1.0) | 96.9 (0.4) | 88.8 (4.9) | 94.7 (3.2) | 97.8 (1.0) | 0.04 µg/L,  0.06 µg/g | ^121^Sb/^115^In,  No gas |  |
| Ba | 74.2 (2.3) | 87.6 (0.9) | 83.7 (16.7) | 70.3 (2.5) | 86.1 (3.1) | 91.3 (0.6) | 61.1 (5.1) | 83.5 (4.3) | 105.3 (0.02) | 1 µg/L,  2 µg/g | ^137^Ba/^115^In,  No gas |  |
| Ir | 110.8 (2.2) | 105.8 (1.9) | 105.9 (2.7) | 111.4^&^ | 105.5 (1.3) | 110.1 (3.4) | 118.2 (4.9) | 108.5 (5.0) | 110.6 (6.3) | 0.004 µg/L,  0.005 µg/g | ^193^Ir/^115^In,  No gas |  |
| Pt | 101.0 (2.1) | 96.7 (1.2) | 93.2 (2.4) | 104.4^&^ | 98.5 (1.1) | 100.5 (2.4) | 111.3 (4.7) | 102.0 (5.4) | 100.8 (6.2) | 0.03 µg/L,  0.05 µg/g | ^195^Pt/^115^In,  No gas |  |
| Au | 136.0 (3.4) | 132.4 (6.5) | 128.9 (1.7) | ND^§^ | 128.8 (0.2) | 131.1 (0.4) | 125.5 (7.5) | 117.2 (6.0) | 117.4 (7.0) | 0.05 µg/L,  0.07 µg/L | ^197^Au/^115^In,  No gas |  |
| Pb | 111.5 (0.2) | 106.0 (0.5) | 98.1 (16.5) | 107.0 (3.0) | 105.2 (0.0) | 109.6 (0.0) | 97.6 (4.4) | 102.8 (2.1) | 106.4 (1.9) | 0.02 µg/L,  0.02 µg/g | ^208^Pb/^115^In,  No gas |  |

^#^ ND: Not detected. V was not detected due to polyatomic interferences. A higher flow of He collision gas could potentially reduce the amount of interference.

^&^ Single determination.

^§^ ND: Not detected. Au was not detected for this sample.
